# Supplementary figures and images for: Resolving the conformational ensemble of a membrane protein by integrating small-angle scattering with AlphaFold
Source: PLoS Comput Biol. 2025 Jun 27;21(6):e1013187. doi: 10.1371/journal.pcbi.1013187 (PMC12251176; doi:10.1371/journal.pcbi.1013187)

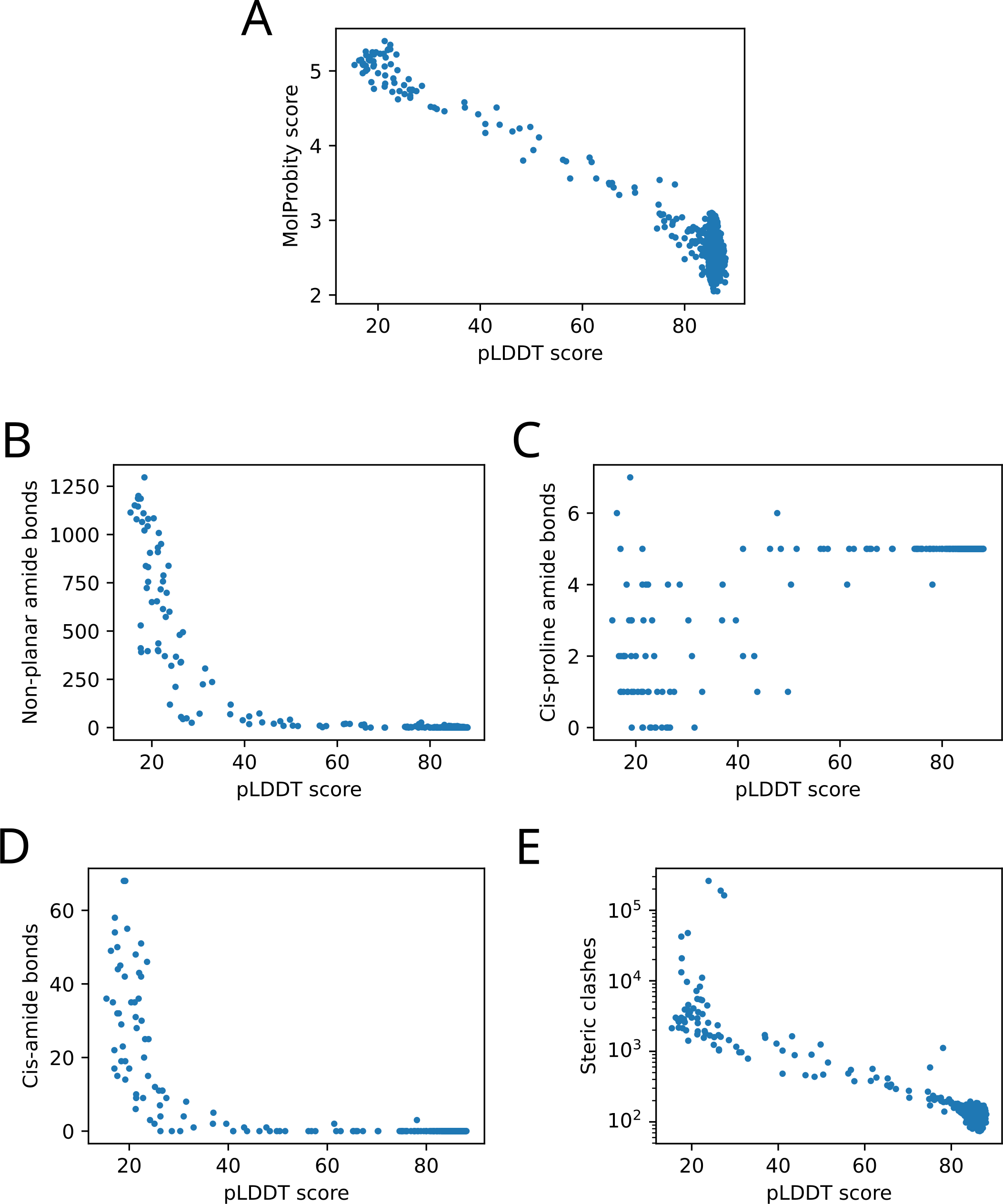

Supplement: S1 Fig — (A) MolProbity scores [29] of all the AF-generated conformations as a function of the corresponding plDDT score. (B, C, D, E) count of (B) non-planar amide bonds, (C) cis proline amide bonds, cis amide bonds and steric clashes, all calculated using the TopModel program [30], for each AF-generated conformation as a function of the corresponding plDDT score. (TIF) [file pcbi.1013187.s001.tif]

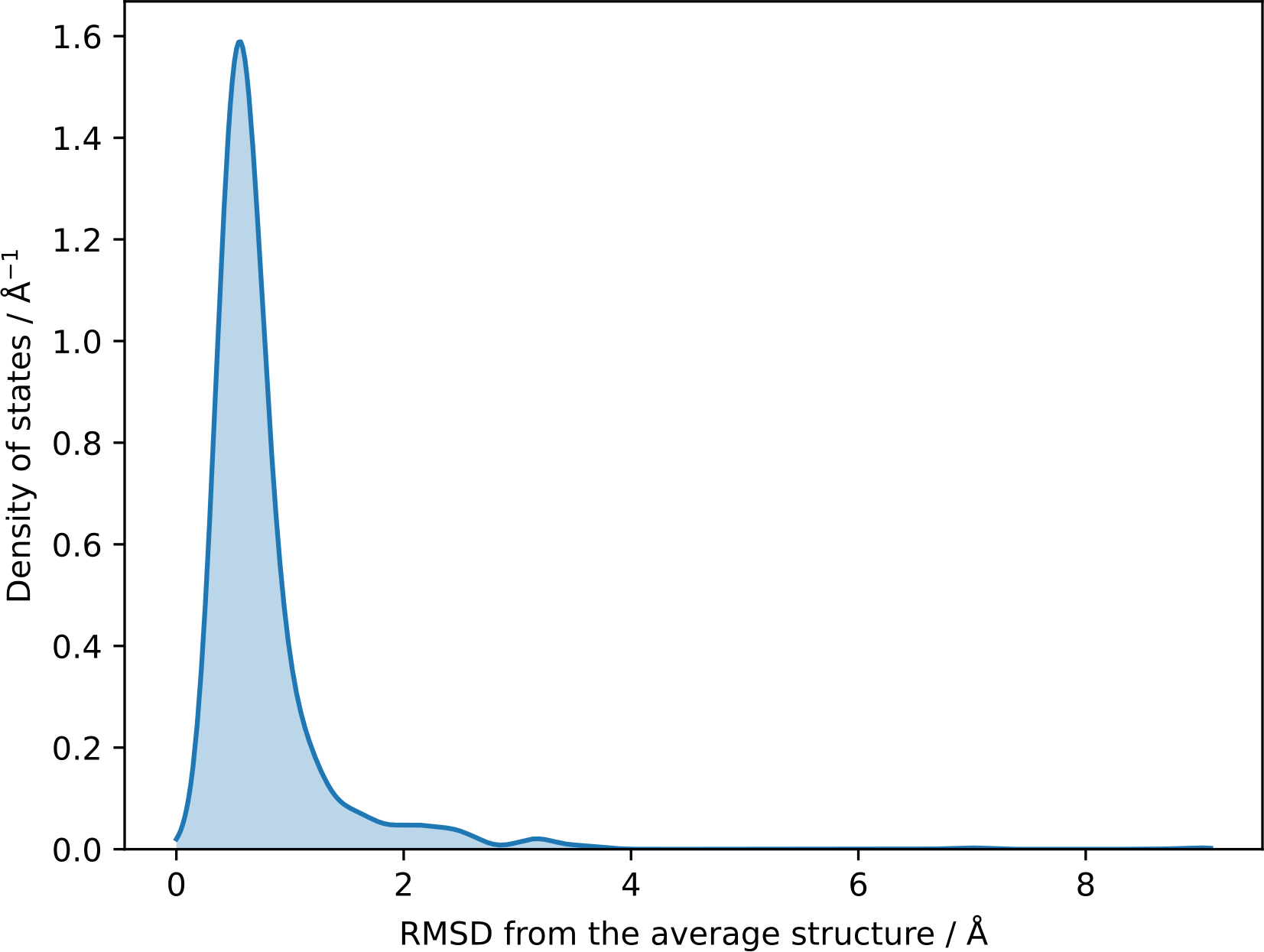

Supplement: S2 Fig — Probability density of the root mean square deviation of the Cα atoms of the AF-generated conformations with pLDDT ≥75, compared to the average structure. Alignment is done on the Cα atoms. (TIF) [file pcbi.1013187.s002.tif]

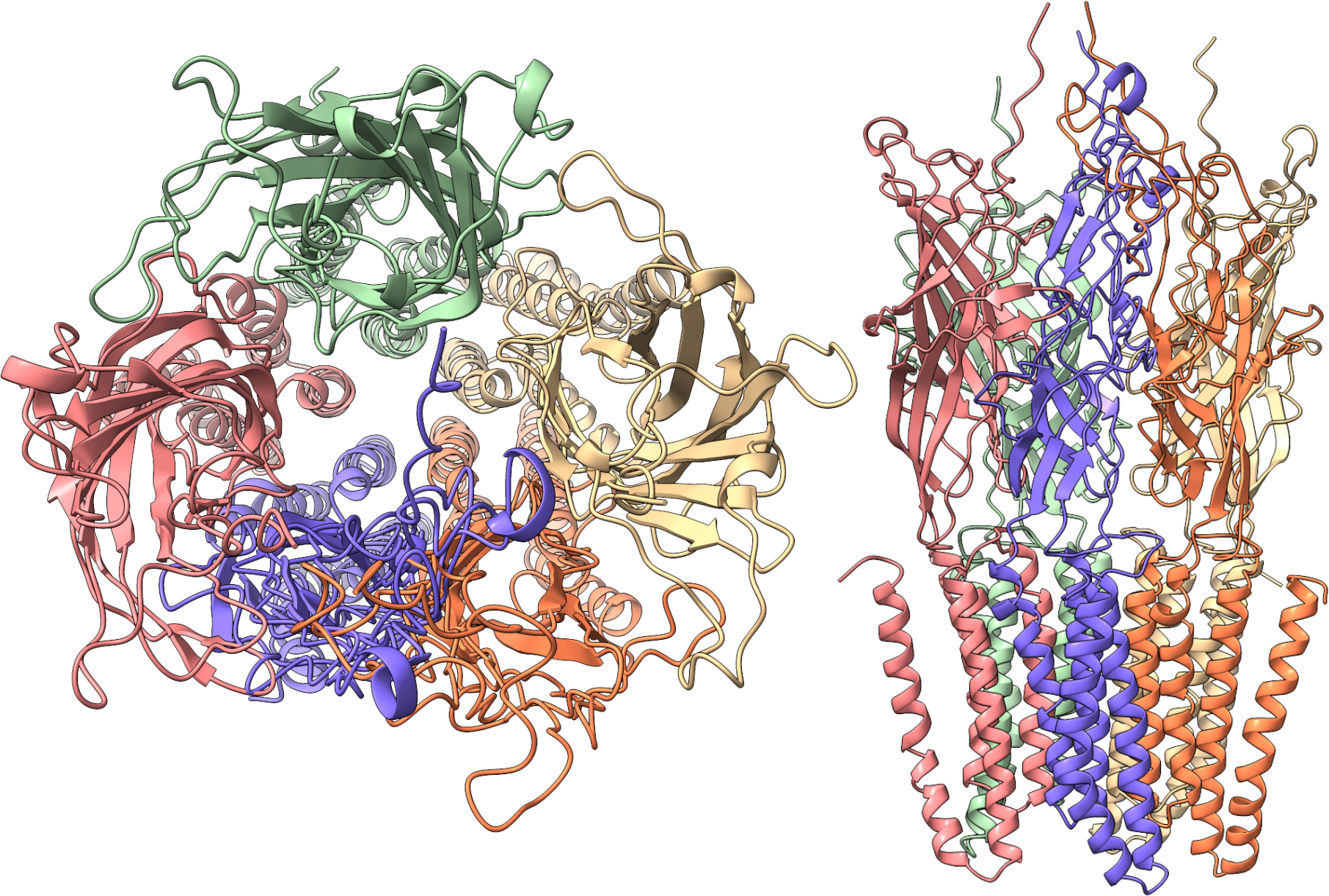

Supplement: S3 Fig — The protein conformation was visualized using ChimeraX [63]. (TIF) [file pcbi.1013187.s003.tif]

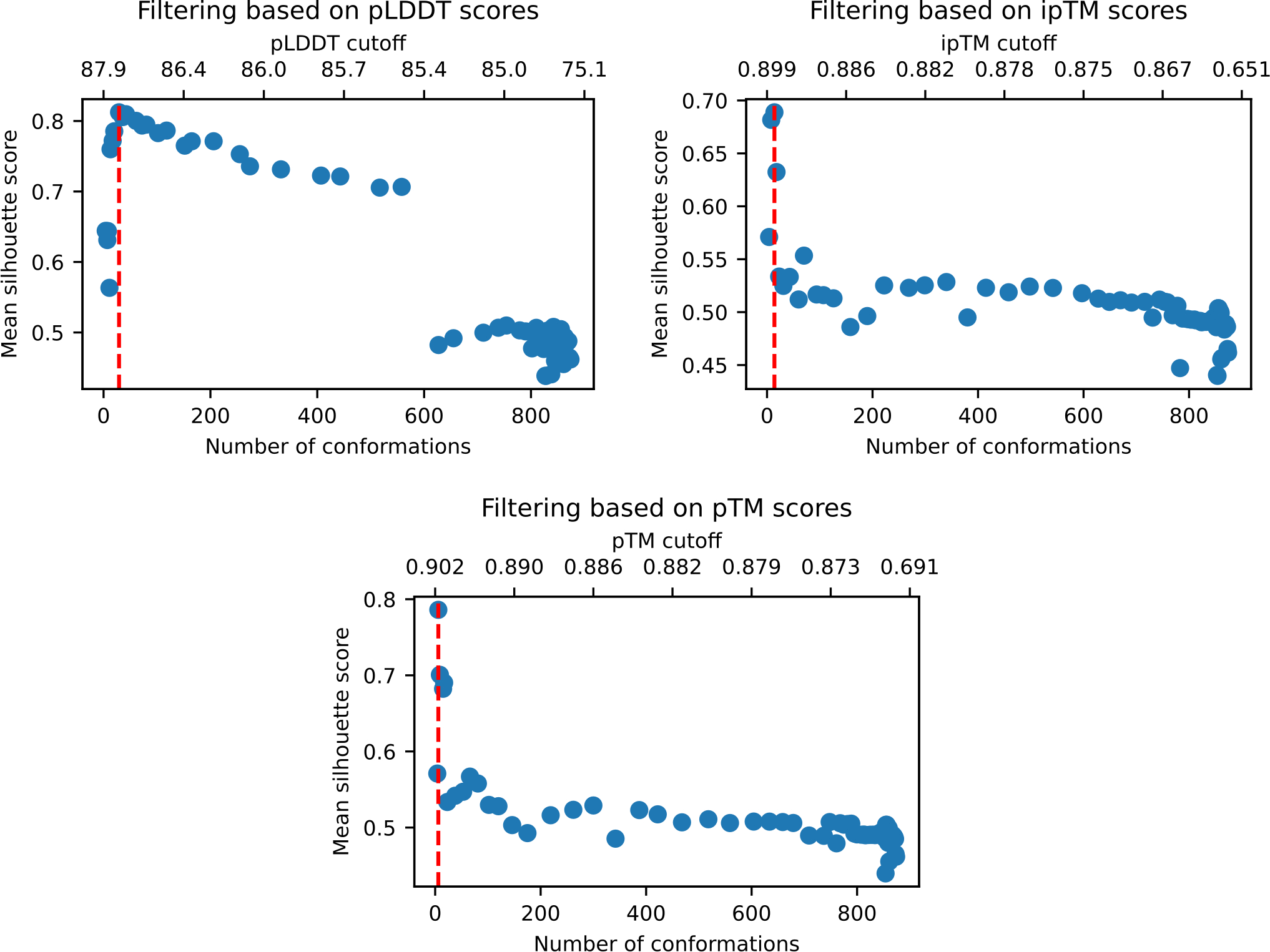

Supplement: S4 Fig — Average silhouette score of the agglomerative clustering of the SANS intensity profiles of all conformations with pLDDT (top left), ipTM (top right), or pTM (bottom) scores above different cutoffs for the initial run of the pipeline, as a function of the number of such conformations. The dashed red line indicates the cutoff for the maximal silhouette score. (TIF) [file pcbi.1013187.s005.tif]

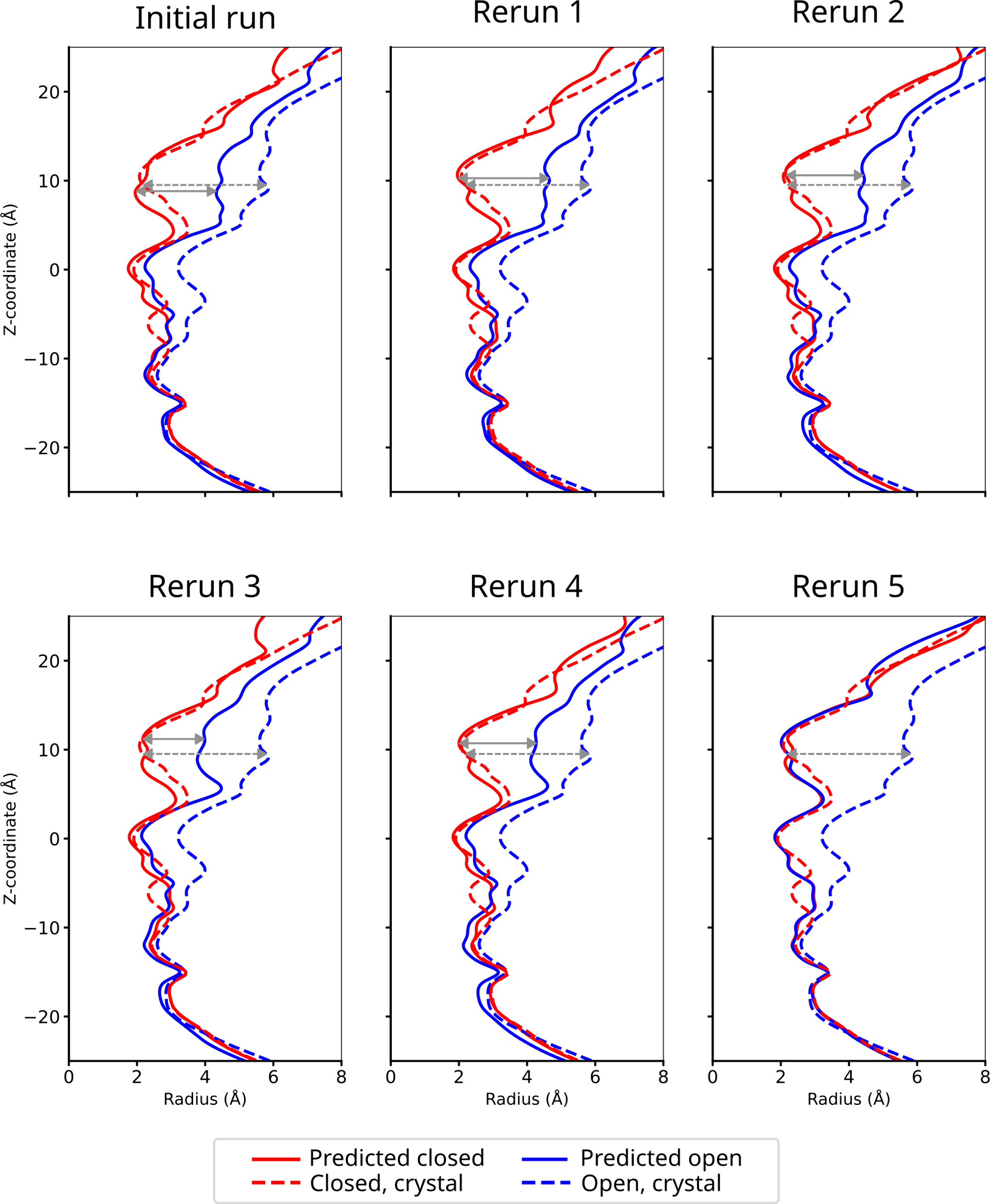

Supplement: S5 Fig — Grey arrows indicate the maximal pore-radii expansions between the predicted conformations (solid) and the crystal structures (dashed). The pore profiles were calculated using CHAP [32]. (TIF) [file pcbi.1013187.s006.tif]

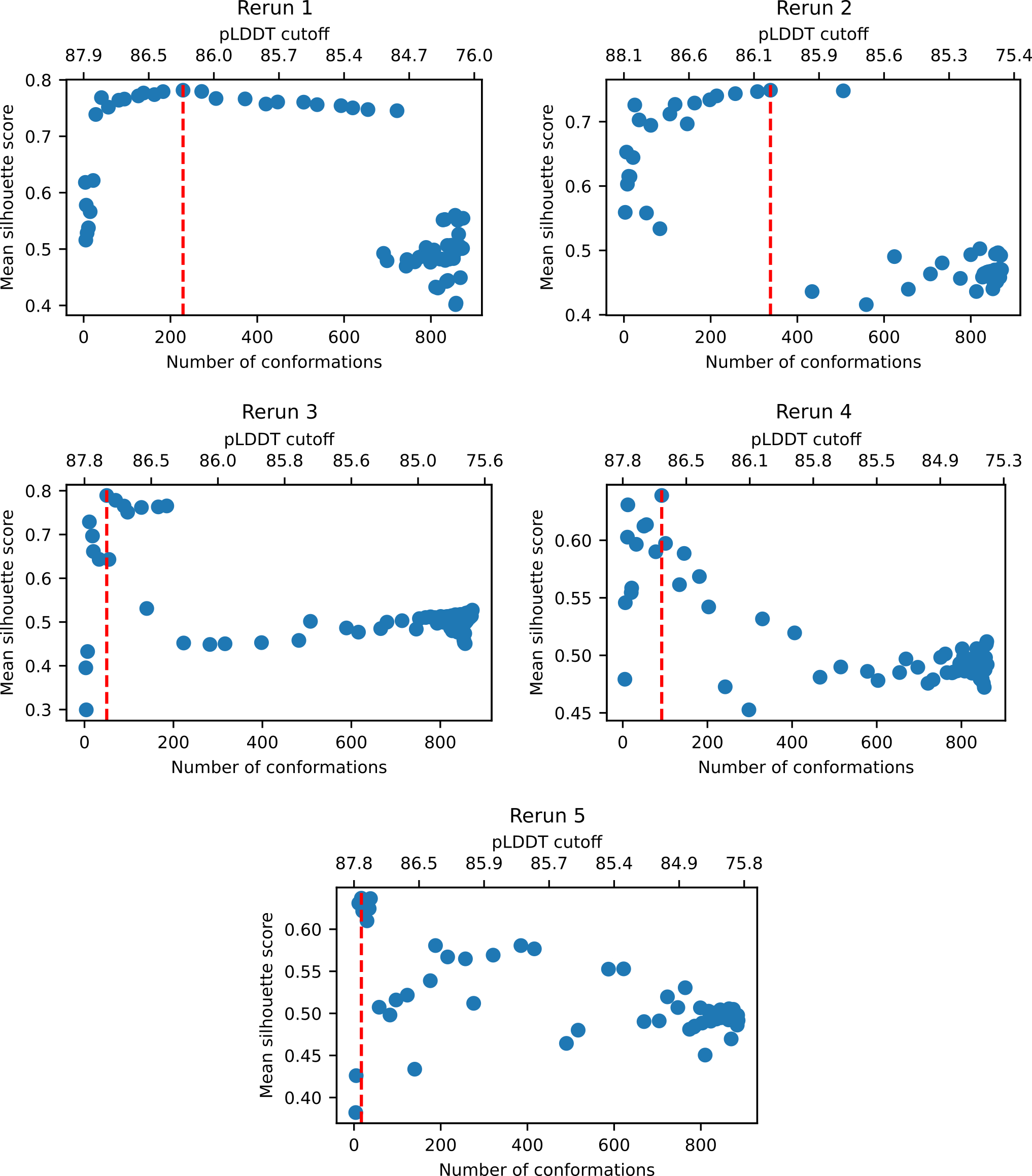

Supplement: S6 Fig — Average silhouette score of the agglomerative clustering of the SANS intensity profiles of all conformations with pLDDT scores above different cutoffs for the five reruns, as a function of the number of such conformations. The dashed red line indicates the cutoff for the maximal silhouette score. (TIF) [file pcbi.1013187.s007.tif]

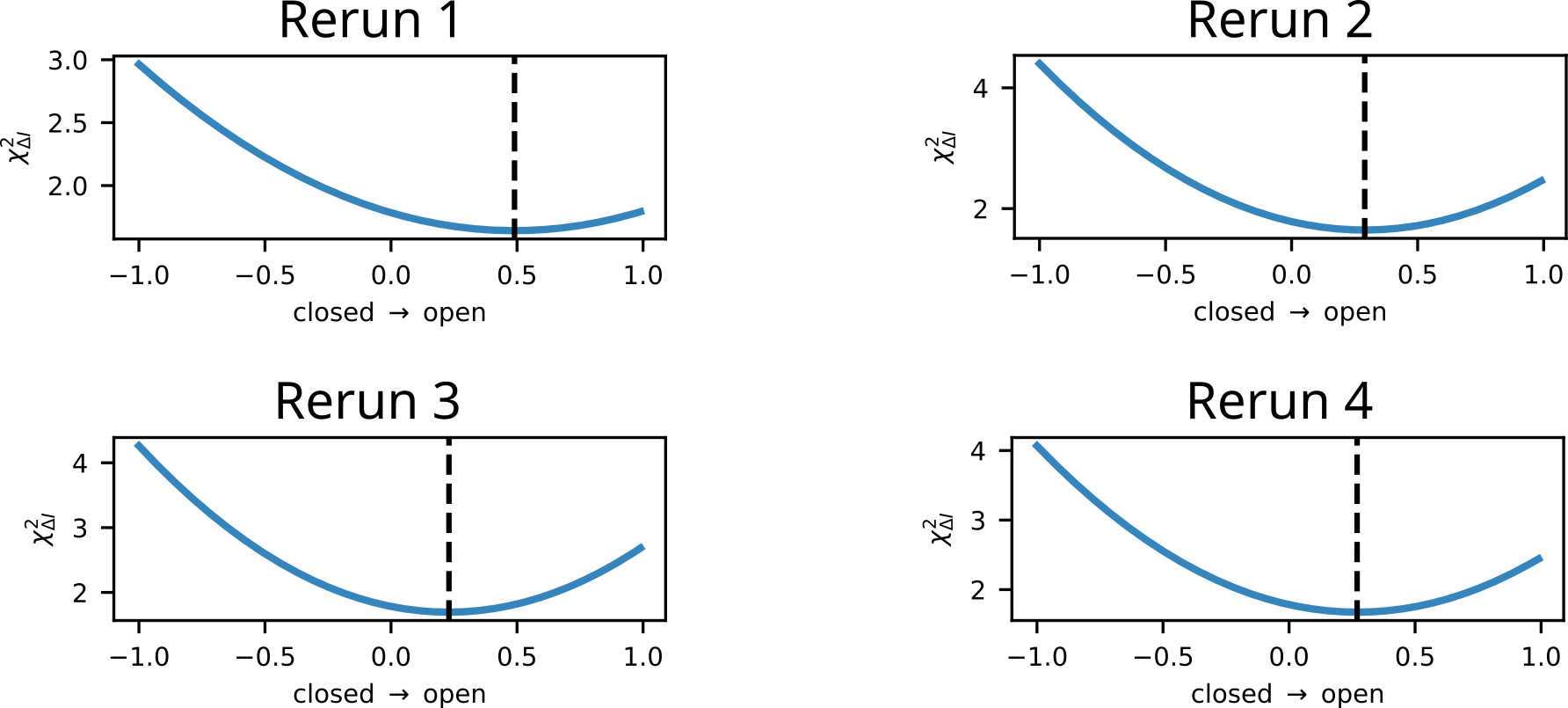

Supplement: S7 Fig — Fit χΔI2 of the predicted intensity difference to the experimental as a function of the population shift from the closed prediction to open prediction, shown for the four reruns in which distinct functional states were predicted. The dashed line indicates the optimal fit. (TIF) [file pcbi.1013187.s008.tif]

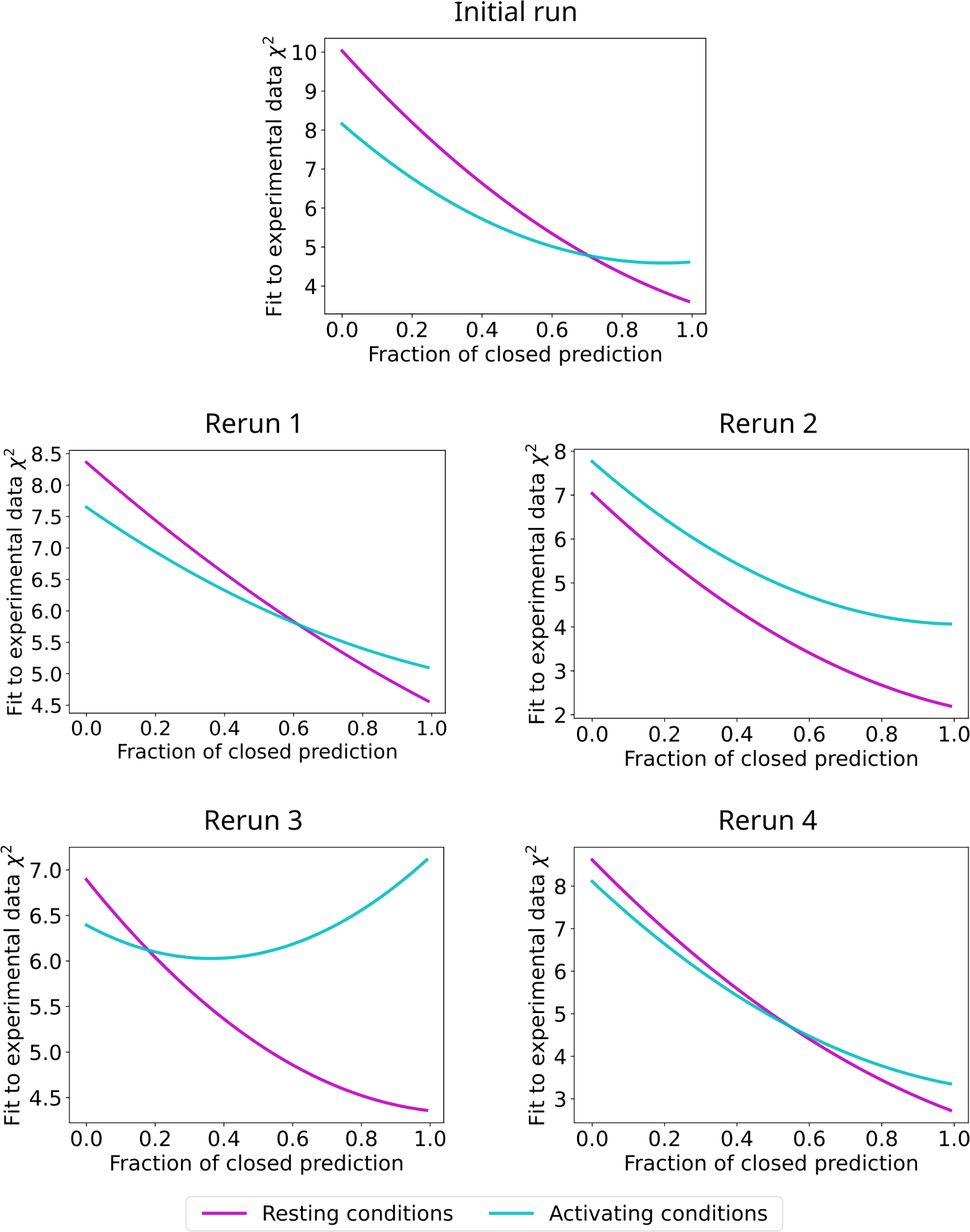

Supplement: S8 Fig — Fits to the experimental SANS data for a linear combination of the closed and open prediction as a function of their relative weights, for the initial run as well as the four reruns in which distinct functional states were predicted. (TIF) [file pcbi.1013187.s009.tif]

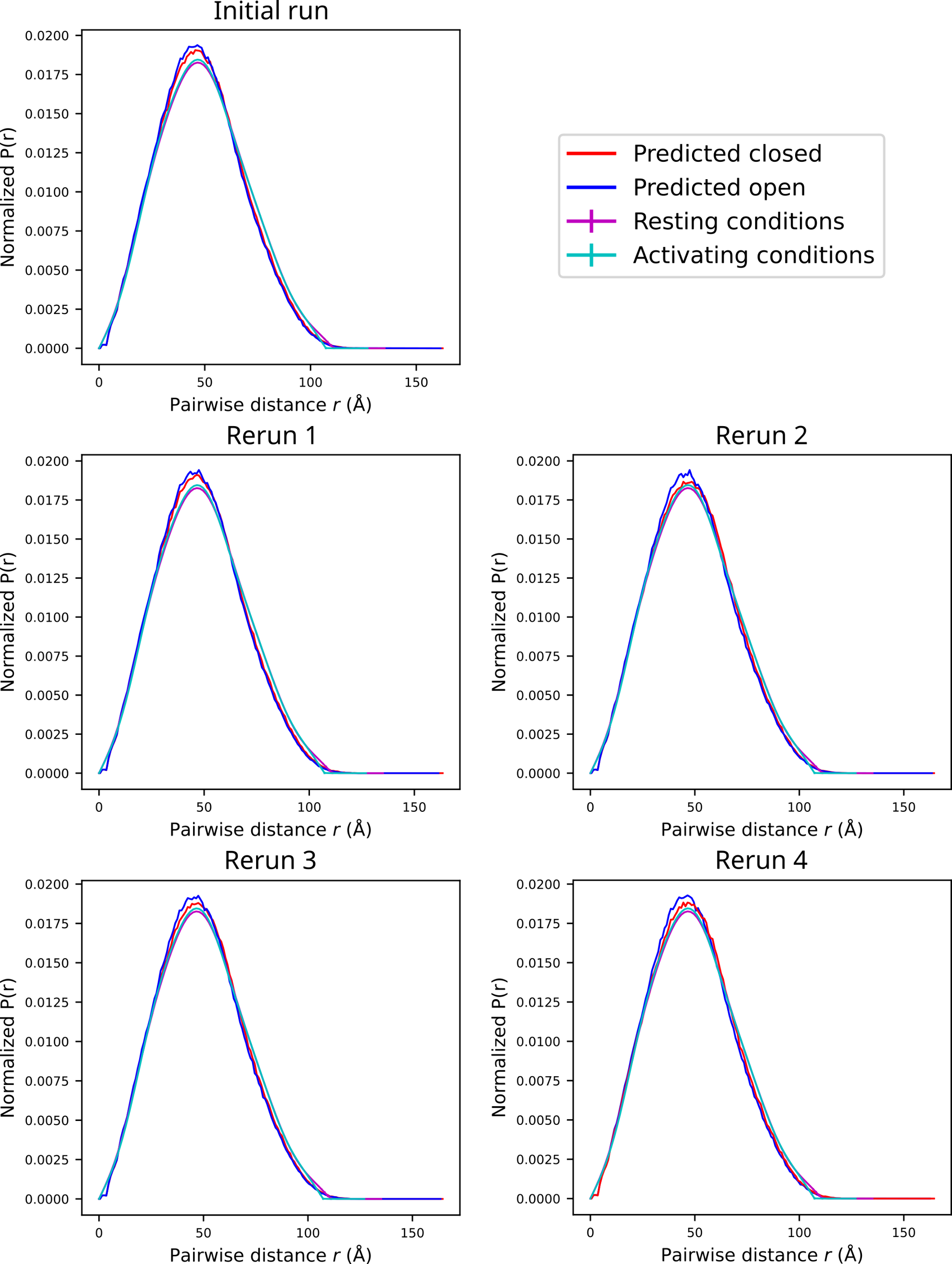

Supplement: S9 Fig — The distributions are normalized by the area under the respective curve. (TIF) [file pcbi.1013187.s010.tif]

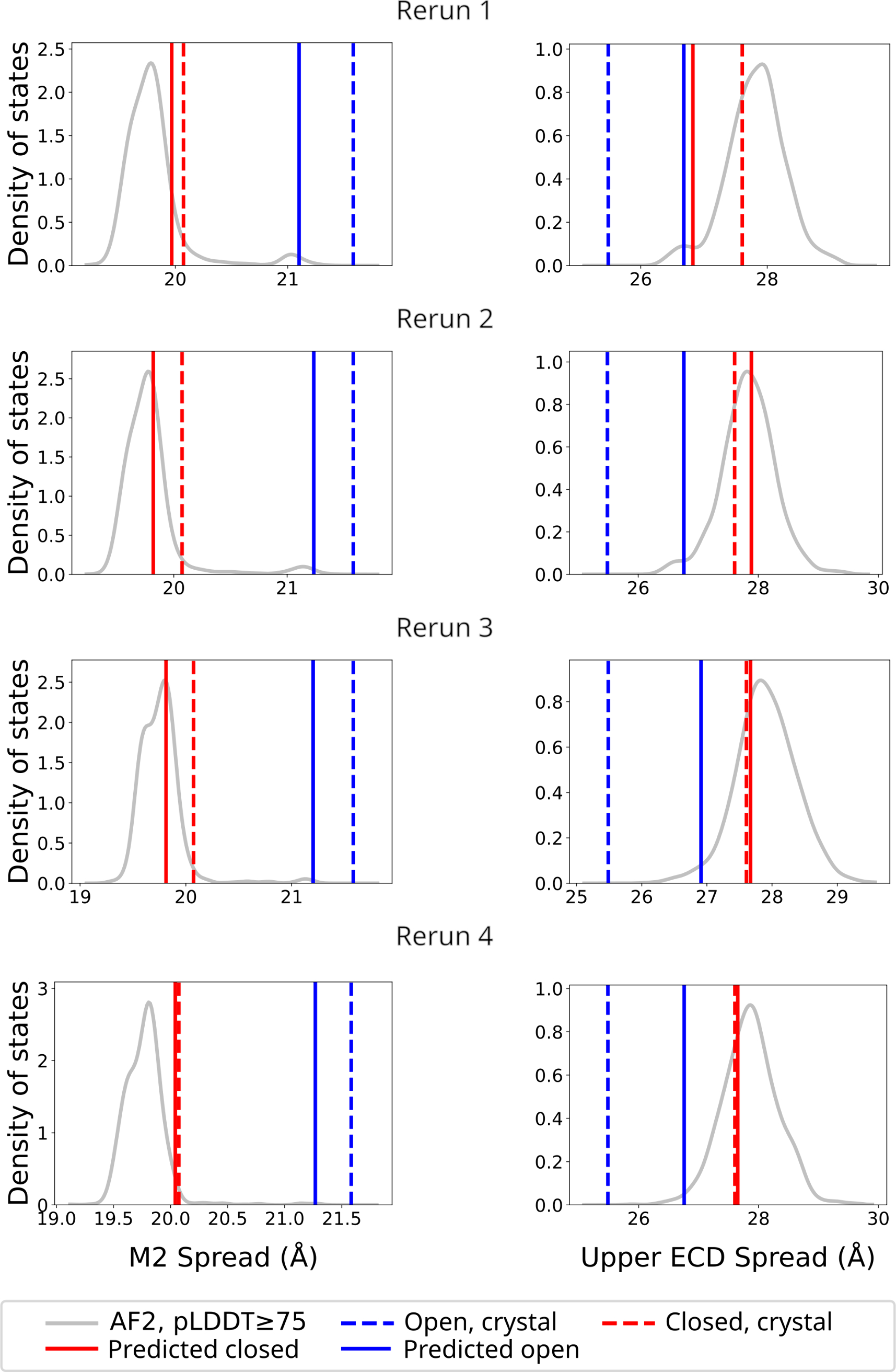

Supplement: S10 Fig — Distance between the centers of mass of the pore and that of the upper part of the pore lining M2 helix (M2 spread) and the upper spread of the extracellular domain (upper ECD spread) for the predicted structures, the crystal structures, as well as the density of states for all AF2-generated conformations with an average pLDDT score above 75. All data is shown for the four reruns in which distinct functional states were predicted. (TIF) [file pcbi.1013187.s012.tif]

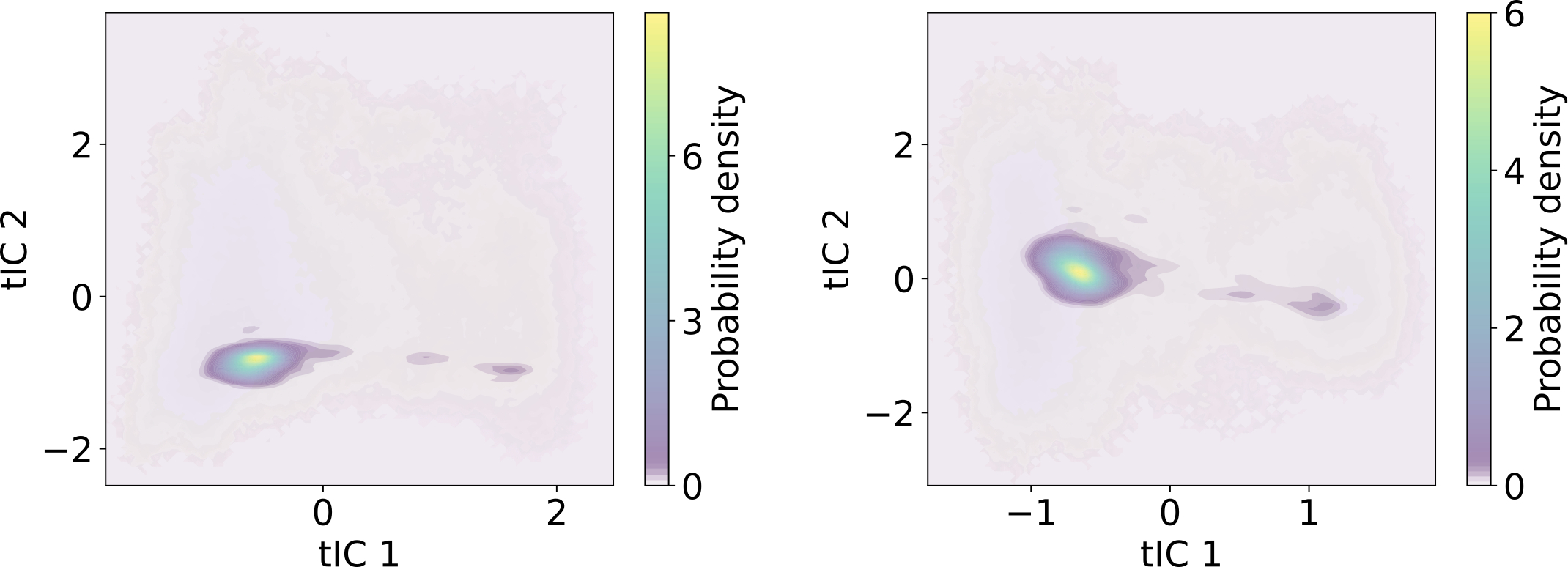

Supplement: S11 Fig — Shown for the initial pipeline run projected onto resting conditions (left) and activating conditions (right). (TIF) [file pcbi.1013187.s013.tif]

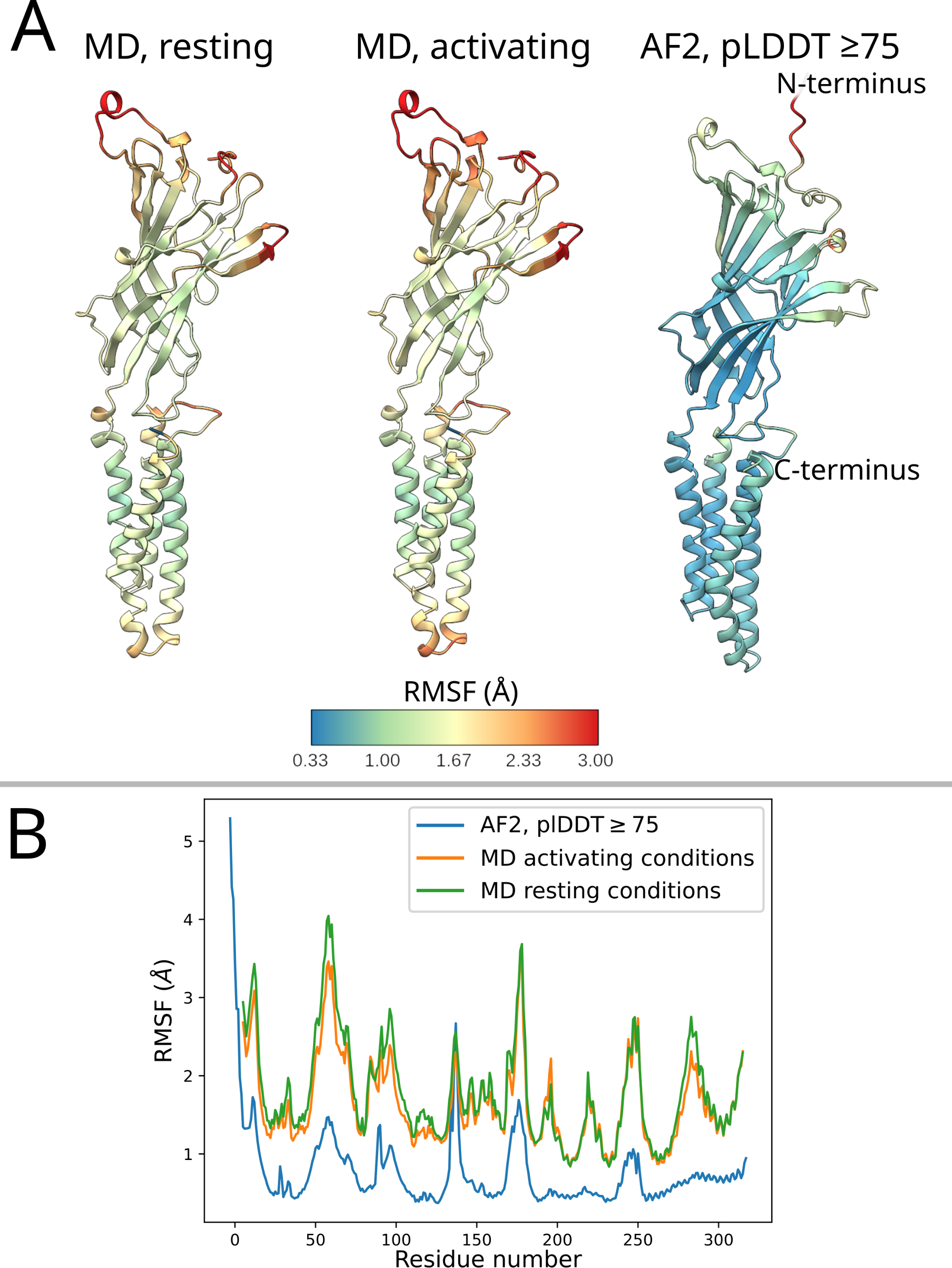

Supplement: S12 Fig — Subunit average of the RMSF for the MD-ensembles as well as the AF-generated conformations (A) projected onto a snapshot from the ensembles and (B) plotted as a function of residue number. The MD-ensembles were retreived from [26], and the molecular models were visualized using ChimeraX [63]. (TIF) [file pcbi.1013187.s015.tif]

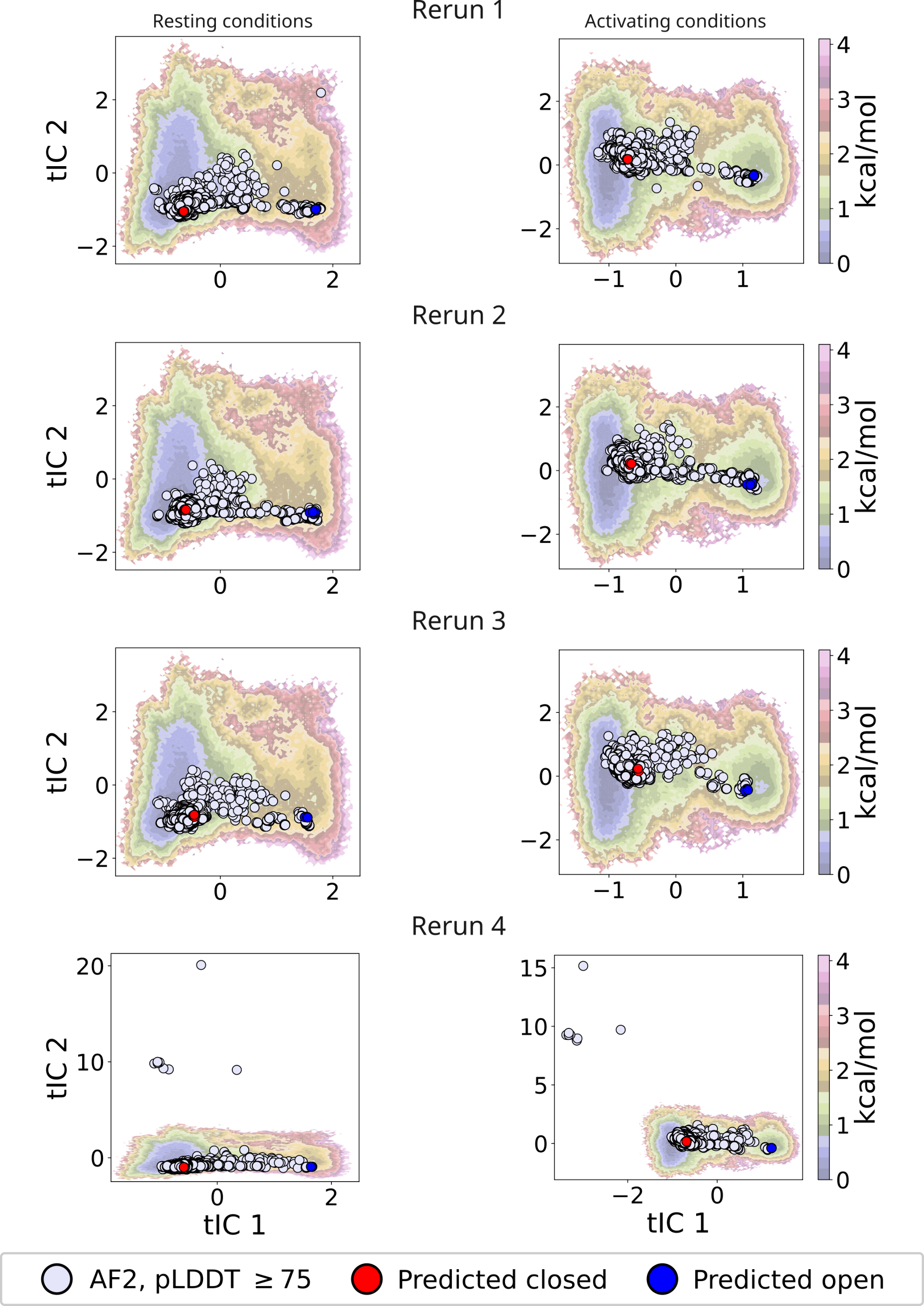

Supplement: S13 Fig — Projections of the AF2-generated conformations with pLDDT ≥ 75 onto the free energy landscape of GLIC at resting and activating conditions, for the four reruns in which distinct functional states were predicted. (TIF) [file pcbi.1013187.s016.tif]
