# Supplementary material for: Resolving the conformational ensemble of a membrane protein by integrating small-angle scattering with AlphaFold
Source: PLoS Comput Biol. 2025 Jun 27;21(6):e1013187. doi: 10.1371/journal.pcbi.1013187 (PMC12251176; doi:10.1371/journal.pcbi.1013187)
Supplement: S1 Table — (PDF) [file pcbi.1013187.s011.pdf]

Data set		Initial run		Rerun 1		Rerun 2		Rerun 3		Rerun 4	
Predicted state		Closed	Open	Closed	Open	Closed	Open	Closed	Open	Closed	Open
RMSD to closed crystal[1] (Å)	All-atom	2.09	2.51	2.02	2.57	1.80	2.36	1.96	2.39	1.9	2.61
	TMD[3]	0.78	1.72	0.59	1.72	0.67	1.69	0.73	1.65	0.61	1.68
RMSD to open crystal[2] (Å)	All-atom	2.02	1.59	2.13	1.63	2.63	1.77	2.45	1.87	2.38	1.80
	TMD[3]	1.56	0.68	1.67	0.66	1.68	0.67	1.63	0.77	1.64	0.76

[a]PDB ID 4NPQ
[b]PDB ID 4HFI
[c]Transmembrane domain
