# Supplementary material for: Resolving the conformational ensemble of a membrane protein by integrating small-angle scattering with AlphaFold
Source: PLoS Comput Biol. 2025 Jun 27;21(6):e1013187. doi: 10.1371/journal.pcbi.1013187 (PMC12251176; doi:10.1371/journal.pcbi.1013187)
Supplement: Attachment — Submitted filename: lidbrink_response_to_reviewers.pdf [file pcbi.1013187.s018.pdf]

Stockholm University

Erik Lindahl
Professor of Biophysics

Stockholm February 25, 2025

Dear Editor,

Thank you for the thoughtful consideration of our manuscript (PCOMPBIOL-D-24-01584). As demonstrated in the attached revision and outlined below, we believe we have responded to all concerns of reviewers 1, 3, and 4, including edits to the Title, Abstract, Methods, Results, Discussion, References, Figures 2 and 3, and new Supplementary Figures S1, S3, S8, S10, S13 and S15. As advised by the editor, we have excluded responses to reviewer 2.

In this response, editor and reviewer comments are gray (renumbered for clarity), and quotes from the revised manuscript are written in red.

Journal Requirements:

J.1. We ask that a manuscript source file is provided at Revision. Please upload your manuscript file as a .doc, .docx, .rtf or .tex.

Response J.1: We have uploaded the unmarked manuscript as a .tex file.

J.2. We noticed that you refer to data points not shown in the legend of S1 Fig. We do not allow references to unshown data, as the PLOS data access policy requires that all data be either published with the manuscript or made available in a publicly accessible database. Please amend the supplementary material to include the referenced data or remove the references.

Response J.2: We have modified the relevant figure (now S2 Fig) to show all data points.

J.3. For main figures, please upload separate figure files in .tif or .eps format.

Response J.3: We have uploaded the figures as .tif files.

Reviewer #1:

Numerous studies have shown that it is sometimes possible to use AF2 to predict conformational ensembles, but selecting functionally relevant states remains a challenge. In this work, Lindbrink and colleagues combine AF2 modeling with small angle neutron scattering data to estimate the relative populations of open/closed GLIC conformations under resting and activating conditions. Their modeling approach aims for rigor and generalizability. Some of the details raise concerns, however.

Major:

1.1. The fits of theoretical curves (generated from AF2 models) to experimental curves do not align very well, especially under activating conditions (chi-squared ~4.5). It is also concerning that fits from replicate AF ensembles varied widely under activating conditions: from 0-64% open. Both the relatively high chi-squared values and the wide variance in results suggests that this form of modeling might not be robust enough to describe the relative populations of open and closed conformations under activating conditions, seemingly one of the main objectives of this manuscript. Could more robust results be obtained if the differences between curves were compared? IE are the relative trends between differences in experimental data and differences in calculated curves were similar, could that provide a more robust result?

Response 1.1: We appreciate the feedback regarding the robustness of population estimates, and agree that they were insufficient. We are especially grateful for the suggestion to compare differences between curves. Indeed, we obtained better χ^2 values and more consistency across replicates by fitting mixtures of theoretical models to the difference between experimental SANS curves. Accordingly, we have added difference-curve analyses to Fig. 3, moved some previous analyses to Fig. S9, and revised Abstract, Results, Discussion, and Methods.

Abstract (p 1):

Stockholm University

Erik Lindahl
Professor of Biophysics

By comparing experimental SANS data under resting and activating conditions, we can quantify the subpopulation of closed channels that open upon activation, matching both experiments and extensive simulation sampling using Markov state models.

Results (p 7):

Given that we were mainly interested in the relative changes between the two states, we focused our analysis on characterizing whether the difference between the predicted states could account for the observed difference between experimental conditions (Fig 3C). An optimal fit to the difference curve between activating and resting conditions ($\chi^2_{\Delta I} \approx 1.6$) was achieved by a 30% increase in the contribution of the predicted open versus closed states (Fig 3D). That is, if the population under resting conditions were entirely closed, the population under activating conditions should be 30 % open. This estimate is consistent with previous reports that a minority population of GLIC transitions from the closed to open state when pH is decreased from 7 to 3 [26, 27, 37].

Fits derived from our additional replicate AF ensembles were comparable to those of the initial test case. Specifically, for the four replicates in which distinct functional states were predicted, the population shift that best fit the difference curve between experimental conditions involved an increase in predicted open versus closed states of 49%, 29%, 23%, and 27% respectively, with $\chi^2_{\Delta I} \in \{1.6, 1.6, 1.7, 1.7\}$ (S8 Fig).

Figure 3 legend (p 8):

(C) Experimental and theoretical SANS difference curves between activating and resting conditions as well as between the open and closed prediction, with the latter scaled to fit the former. An error-normalized residual is shown below. (D) Fit $\chi^2_{\Delta I}$ of the predicted versus experimental difference curves as a function of the population shift from the closed prediction to open prediction. The dashed line indicates the optimal fit, and corresponds to a 30% increase in the contribution of the predicted open versus closed states.

Discussion (p 11):

The subtle gating transitions and a low open probability of GLIC only resulted in minor differences in the experimental SANS curves [27], but we could still robustly estimate the population shift between the experimental conditions that largely agreed with expectations (Fig 3).

Methods (p 14):

To compute the change in experimental intensity for some scattering vectors q that differed slightly ($<10^{-4}$ Å^{-1}) between the experimental conditions, we linearly interpolated the experimental data under activating conditions to match the scattering vectors under resting conditions, with error bars determined through error propagation.

For the predicted change in SANS curves we used the average $\underline{f}$ of the scaling parameters f that minimized Eq. (1) for the activating and resting conditions, and computed the fit $\chi^2_{\Delta I}$ of the predicted vs. experimental difference curves as

$$\chi^2_{\Delta I} = \sum_{i=1}^{N_q} \frac{[\underline{f} \cdot (\delta w\, I_1(q_i) - \delta w\, I_2(q_i)) - (I_{exp}{}^{active}(q_i) - I_{exp}{}^{resting}(q_i))]^2}{N_q \cdot (\sigma^2{}_{active}(q_i) + \sigma^2{}_{resting}(q_i))}, \quad (2)$$

where δw signifies the change in weights from prediction 2 to 1, $I_{\mathrm{exp}}{}^{\mathrm{active}}$ and $I_{\mathrm{exp}}{}^{\mathrm{resting}}$ the experimental SANS intensities under activating and resting conditions respectively, and σ_{active} and $\sigma_{\mathrm{resting}}$ the corresponding experimental errors.

1.2. The efficiency of using AF2 over MD simulations is appreciated. Still, how do the fits of low-energy MD conformations compare with SANS experiments, and what does that say about the

Stockholm University

Erik Lindahl
Professor of Biophysics

Stockholm February 25, 2025

usefulness of AF2-based modeling? This question seems especially important because the predicted closed AF2 structures do not appear to overlap with the energy minima in Figure 5.

Response 1.2: We thank the reviewer for highlighting this important point; indeed, even AF models filtered for high pLDDT scores are not expected to recapitulate free-energy minima as effectively as thorough MD simulations. We have modified the Results, Discussion, and Methods to expand upon it.

Results (p 7):

We next examined the direct fits to the experimental SANS curves, which provide an additional way of estimating the state populations. For all relevant replicates, the best fit to resting conditions (with $\chi^2 \in \{3.6, 2.2, 2.7, 4.4, 4.5\}$) was achieved by the predicted closed conformation, while the best fit to activating conditions (with $\chi^2 \in \{4.6, 6.0, 3.3, 4.1, 5.1\}$) was achieved by linear combinations of 92/8 %, 36/64 %, 100/0 %, 100/0 %, 100/0 % closed/open conformations (S9 Fig, Fig 3C, D). To characterize the quality of these predictions, we sought to compare them to approximate ground truth models derived from extensive molecular dynamics (MD) simulations [26] (described in more detail below). We found that the MD-sampled conformations representing the closed-state basin individually fit the experimental SANS data slightly better, with χ^2-values of 2.0 ± 0.3 (mean ± standard deviation) under resting conditions and 2.0 ± 0.5 under activating conditions, as determined by the implicit solvent method Pepsi-SANS [31].

Discussion (p 12):

Our predicted open and closed models were consistent with previously reported X-ray structures, and approached corresponding basins in free-energy landscapes from our previous extensive MD simulations [26]. Still, MD-sampled conformations representing the closed-state basin fit the experimental SANS data slightly better than our AF-based models. It is perhaps unsurprising that models derived from extensive statistical sampling of MD simulations are somewhat better optimized to solution-phase structural data than AF2 models, which may be influenced by contact optimization and/or structural training data. With machine learning algorithms rapidly improving, the potential loss in accuracy is constantly decreasing and in particular, given the dramatic reduction in computational cost, it should be possible to compensate by simply increasing sampling or using short simulations to improve occupancy predictions after the initial ensemble prediction.

Methods (p 14):

SANS curves of MD-sampled closed-state representatives

To select representative conformations of the closed-state basin, we randomly sampled 1550 conformations from the resting (deprotonated) MD ensemble [26] and chose the 277 conformations that had free energies below 0.5 kcal/mol. The corresponding SANS curves were calculated using (the implicit solvent method) Pepsi-SANS [31], with $I(0)$, $\delta\rho$ and r_0 optimized for the best fit to the respective experimental curves.

1.3. The clustering approach by plDDT seems a bit overdone. Looking at Figure 2A, by eye, there seem to be 3 clusters (a large one centered around 0.0, a second centered on 0.4, a third on 0.6). There does not seem to be a strong justification to increase plDDT threshold, especially given the next comment.

Response 1.3: We regret failing to fully justify the pLDDT threshold applied. To illustrate the need for this extent of quality filtering, we have introduced a new SI figure (S3 Fig.) showing that some structures with average pLDDT scores above 75 still can be partially misfolded. For clarity and consistency, we have also updated Figure 2A to show the PCA for a pLDDT cutoff of 75 instead of 80. We also elaborate on the rationale for increasing the cutoff in Results (p 5):

Stockholm University

Erik Lindahl
Professor of Biophysics

133 conformations (15 %) deviated more than 1 Å from the average structure, and 17 deviated more than 2.5 Å (S2 Fig), suggesting that multiple functional states might be represented. Still, some members of even this filtered ensemble featured implausible geometry (S3 Fig). We determined that an alternative analysis was needed to appropriately filter nonphysical structures and distinguish plausible functional states.

To this end, we assessed our ensemble of AF-generated models according to low-resolution structural information accessible by SAS. Since SAS data cannot be inverted to directly predict structures, we instead predicted theoretical curves for all GLIC conformations in our ensemble, using the implicit solvent method implemented by Pepsi-SANS [31]. By performing principal component analysis (PCA) of these profiles, and projecting onto the first two principal components (PCs) (which accounted for more than 95% of the total variance), this initially filtered ensemble appeared to represent a single cluster (Fig 2A). However, by progressively restricting the number of conformations in the PCA by raising the pLDDT cutoff, we found that the data increasingly split into two clusters (S4 Video). We hypothesized that distinct clusters could correspond to distinct physiological states of GLIC, and sought to find an appropriate pLDDT score cutoff. For this purpose, we performed agglomerative clustering for different cutoff values and calculated the average silhouette scores of the clusterings (see Materials and methods for details). The average silhouette score increased from 0.46 (indicating poor cluster separation) for the initial pLDDT cutoff of 75, to a maximum of 0.79-0.81 (reflecting more distinct cluster separation) for pLDDT cutoffs of 86.5-87.2 (S5 Fig). At the latter cutoffs, two distinct clusters were visually clear (Fig 2B), and we selected the conformations with the highest average pLDDT score in each cluster as the best-quality models.

1.4. Several recent papers have shown that plDDT scores do not necessarily discriminate between correct and unphysical AF2 predictions (PMIDs: 37981824, 39181864, Bryant and Noé, Nat. Comms. 2024). Since the work in this article separates different conformations by plDDT (Fig 2), can the authors comment further on when the method they propose is likely to be successful and when it may not be?

Response 1.4: pLDDT is definitely not a perfect classifier even just for screening, and it might not be suitable for all systems. We include movies with several other AF metrics in the SI, and have made the following changes to the Discussion (p 11):

Some analytical choices in this work, for example in the clustering of SAS curves and selection of representative conformations, may be system-dependent. In the case of GLIC, SANS curves predicted for our AF-generated models consistently formed two distinct clusters once filtered by an appropriate pLDDT cutoff, where intermediary states had been filtered out. However, average pLDDT scores are thought to indicate general model quality, not necessarily thermodynamic stability [44]. Moreover, a recent study has shown that pLDDT scores can be biased toward structures already published in the PDB, and may not correlate strongly with model quality for alternative conformations [45]. The usefulness of pLDDT as a screening metric may thus be system-specific. One approach to tackle this is to try multiple AF metrics like pLDDT, pTM and ipTM, as well as structure quality metrics like MolProbity, and select empirically a filtering protocol that identifies distinct clusters. Alternatively, it may be more useful to filter conformations to an acceptable level of geometric plausibility, and then choose representative conformations that are distinct in the SAS PCA space, even if intermediary conformations are still present.

Stockholm University

Erik Lindahl
Professor of Biophysics

Stockholm February 25, 2025

Minor:

1.5. Another manuscript (Riccabona, et al bioRxiv 2024) suggests that AF2's ability to replicate MD ensembles is limited. How can that be reconciled with the results presented here?

Response 1.5: While we were able to sample some GLIC conformations in both the open and closed energy basins, we indeed found that this sampling does not replicate the MD *ensemble distribution*. To highlight this (also in response to comments 3.4, 4.1, and 4.3), we now compare the root mean square fluctuation of the AF-generated ensemble to the MD ensembles in a new supplementary figure (S15 Fig), and have modified the Results and Discussion appropriately.

Results (also in response to 4.1) (p 10):

It is not certain that transitional conformations in tIC space represent functional transition states, as they could deviate along other degrees of freedom orthogonal to the first two components. Still, particularly in light of its narrow RMSD range, our AF-generated ensemble appeared to follow the predicted transition path, with spreads corresponding to the wells and saddle points in the free-energy landscape (Fig 5). MD-generated ensembles sampled generally greater root-mean-square fluctuations (RMSFs) than our AF ensemble, particularly in loops between strands or helices; still, the profile of low versus high flexibility was consistent between methodologies (S15 Fig).

Discussion (also in response to 3.4 and 4.3) (p 12):

Here, we represented the conformational ensemble of GLIC using only two states. This is an oversimplification, and given the imperfect fits to the SANS data, it might be appropriate to include more AF-sampled states. Indeed, our AF ensemble sampled a similar pattern of RMSF as our previous MD-generated ensembles (S15 Fig); on the other hand, it did not replicate either the density of states or quantitative flexibility (Fig 5, S13 Fig). Since AF sampling is not statistically representative, it is not straightforward to include more conformational states without running the risk of overfitting to noise in the SANS data. To attain a Boltzmann-distributed ensemble of protein states, one approach could instead be to use the AF-sampled conformations as seeds for MD simulations e.g. in line with [24]. Since the AF-sampled states seemingly ranged continuously from open- to closed-like for GLIC (Fig 5), it is plausible that simulations starting from these seeds could reconstruct the free-energy landscape.

We now also comment on that MSA-subsampling might fail to capture alternative states for other proteins in Discussion (p 11):

Still, MSA-subsampling might fail to sample alternative states for other proteins (e.g. as seen in [46]).

Reviewer #3

This paper, by Lidbrink and colleagues, tackles the complex problem of predicting distinct conformations of membrane proteins involved in signaling. It is an important problem whose solution would benefit multiple areas of health research. The authors innovatively combine a Jens Meilleur program to generate a diverse conformational set with their previously published experimental SANS data. They show that use of PCA analysis, they can generate models consistent with crystal structures of open and closed states. There is concern for potential "cheating" by AF3 with already deposited PDBs. The larger concern is that they state that their models are consistent with resting and activated states measured by SANS, which is not readily apparent in their figure. See discussion below.

3.1. I understand the part of using MSA sampling to generate different ensembles. On page 4 line 76, authors determined to remove conformations with a plDDT lower than 75. Are there any

Stockholm University

Erik Lindahl
Professor of Biophysics

specific reasons for the cutoff? Will this cutoff remove flexible conformations from the prediction?

Response 3.1: We agree that the cutoff might have come across as arbitrary, and have now (also in response to 4.1) added a supplementary figure (S1 Fig.) highlighting poor geometric properties of the conformations with pLDDT scores below 75, and updated the Results (p 4-5):

We observed that conformations with average predicted local distance difference test (pLDDT) scores [17, 28] < 75, in general, had poor structural quality when assessed by metrics such as MolProbity score [29] and the number of steric clashes and cis-amide bonds [30] (S1 Fig). Therefore, as an initial quality filter, we removed all conformations with average pLDDT scores below 75, leaving 874 conformations.

3.2. The authors state that their generated state assignments are consistent with the respective excited or resting state experimental data. From first glance at Figs. 3A and 3B, the models are consistent with the overall features of the experimental data. Yet, the goal of this work is to generate atomic predictions for individual states. Given that rigorous criteria, it is difficult to judge that the open or closed models agreed better to the experimental data than the other. Their residual shows a clear, q-dependent bias, indicating a significant difference between experimental and the atomic models. In this case, use of chi square could hide discrepancies. Could the authors explain based on the residuals how they reached their conclusion? Comparing experiments with models in both reciprocal and real space plots can give distinct information and provide more intuitive insight into the difference.

Response 3.2: We agree that the residuals indicate imperfect fits, and after suggestions from reviewer #1, we performed a new set of analyses focusing on the change in SANS intensity. We believe this analysis provides significantly more robust results, and have thus renamed the subsection and figure to "Change in experimental SANS profiles corresponds to the difference between generated states"; see response 1.1 and Results (p 7) for relevant text changes.

We have added a new supplementary figure (S10 Fig) with real space plots, and comment on the imperfect fits in the same subsection of Results (p 7):

To investigate the discrepancies in the fits from the AF-sampled conformations, we calculated the corresponding residuals which exhibited a clear q-dependent bias, as seen in (Fig 3A, B). For a more intuitive view, we compared the experimental SANS data and the predicted intensities in real space (using the tool "calculating pair distance distribution functions for proteins", CaPP) [38]). Both the predicted models appeared to be modestly contracted relative to experimental SANS profiles (S10 Fig). Interestingly, the population estimates based on the experimental difference curves appeared to be less affected by these discrepancies than the direct fits.

3.3. For the experimental data, 1 or 2 sentences on how the data was collected would be relevant for this paper for assessing the efficacy of the methods. For example, was any size exclusion performed. How long was the data collection? What temperature? Concentration of ligand.

Response 3.3: We have now expanded upon the experimental data collection when introducing it in the Results section as follows (p 6-7):

These results were compared to experimental SANS curves for GLIC collected using a paused-flow size-exclusion chromatography setup under pH 7.5 (resting) and pH 3.0 (activating) conditions, collected at 10° C for 58 and 53 min respectively with average concentrations of 0.77 and 0.74 mg/ml, respectively [27] (Fig 3A, B).

3.4. In Fig.3C, the authors try to use a linear combination of closed and open states to evaluate the prediction accuracy. Since a conformational ensemble usually does not consist of only two distinct conformations, is there a better way to evaluate the prediction result instead of simply

Stockholm University

Erik Lindahl
Professor of Biophysics

using a linear combination of two PDBs? The authors did pathway analysis – could models along the pathway be used?

Response 3.4: While we noted that some conformations seemed to project onto the transition pathway on the MD-generated free energy landscapes, we did not perform any pathway analysis. We agree with the reviewer that the two-state model is an oversimplification, also emphasized in comments 1.5 and 4.3; however higher-energy conformations should have very low population at equilibrium, and since the AF2-generated models are not statistically representative, ensemble fitting is not straightforward. We now expand upon this point; see response 1.5 and Discussion (p 12) for details.

3.5. Additionally, it is the ensemble of closed and open structures that should be shown in comparison to the experimental data for Fig 3A.

Response 3.5: We thank the reviewer for the suggestion, and we computed the ensemble average SANS curves of the 2 clusters from Figure 2B, shown below. However, we believe there are several reasons why including this analysis in the manuscript may not be appropriate. First, the AF-sampling is not a statistically representative distribution, and the clusters and the resulting ensemble curves become sensitive to the exact pLDDT value used. Second, the SANS curves below are calculated using the implicit solvent method in Pepsi-SANS, since the explicit solvent method becomes overly cumbersome for a large number of conformations; therefore, the closed and open curves for the single predictions deviate slightly from the corresponding explicit solvent curves in the main text, which may create confusion. Since the ensemble curves are highly similar to the respective prediction curves, and our main goal is to find representative conformations of both states, we hope that the reviewer agrees with our choice to focus the manuscript on the existing analyses.

3.6. The authors discussed the comparison between PDBs and predictions In the section starting from page 7 line 167. Clearly, there are some differences including the distribution of closed/open states and Fig.4 F. I suggest the author to further explain those differences. What does it mean that the proteins are primarily in the closed state?

Response 3.6: This is an interesting point. While it would be tempting to say the predictions reproduce the closed state having lower free energy (since only a minority of channels are open even under activating conditions), that is likely hard to justify based just on a single example, but it is reassuring we are not biased by the structures in the PDB. Following the reviewer's comment, echoed in comment 4.2, we now expand upon this in the Discussion (p 11):

In the case of GLIC, it was possible to sample both open- and closed-like states. Interestingly, AlphaFold sampled mostly closed-like conformations (Fig 4, S13 Fig), even though a substantial majority of GLIC structures in the PDB are open [41]. This suggests that the distribution of AF-generated conformations is not heavily biased by the distribution of structures in the PDB. Instead, the distribution might depend on the structural details of the different states like the number of contacts formed, suggesting that this method is generally applicable even when one state is unknown.

3.7. The application of this method is on proteins with unknown structure. How would AF be influenced by structures already in the PDB, even if use of template is blocked? Has this been established for this work?

Response 3.7: We appreciate this point, also highlighted by reviewer 1, and have modified the text accordingly. See response to 1.4 and Discussion (p 11) for details.

3.8. Do the models have proper geometry, based on Jane Richardson's program Molprobity? If they do not, this can impact comparison to experimental data.

Response 3.8: As described above in response 3.1, we now calculate and present the MolProbity score of all AF2-generated conformations in a new supplementary figure (S1 Fig).

Stockholm University

Erik Lindahl
Professor of Biophysics

Stockholm February 25, 2025

Reviewer #4

The authors address a very important question in the field, namely how to combine AF-subsampling with experimental data and how well the subsampled conformational states align with extensive MD data.

I think the story is well written and the analysis is timely. I have three remarks that should be addressed before publication.

4.1. First, the authors mention that they excluded (quality filtered) structures based on low pLDDT scores. Another way to check if conformations are plausible are to check for D-amino acids/cis-amid bonds or sterically clashes. Did the authors consider to check for those inaccuracies? I would be curious to see whether low pLDDT scores correlate with physically incorrect/implausible structures? (https://www.tandfonline.com/doi/pdf/10.1080/19420862.2023.2175319, https://www.nature.com/articles/s42003-023-04927-7), also in line with that, did AF subsampling pick up areas of highest flexibility compared to the MD? Could the authors use in addition to the tICA like a B-factor plot overall comparing the subsampled ensemble with the MD ensemble to compare areas of flexibility?

Response 4.1: Appreciated. We now report the above-mentioned geometric scores in the new S1 Fig.; see response 3.1 and Results (p 4-5) for details. We also compare the RMSF in our AF-generated ensemble with MD ensembles in S15 Fig.; see response 1.5 and Results (p 10).

4.2. Also did the authors observe if the relative predicted populations resulting from subsampling correspond to the number of structures available in the PDB of the respective states?

Response 4.2: Good suggestion. We now expand on our previous ensemble characterizations regarding M2 and ECD spread, now also quantifying the population of open versus closed states in relation to the free energy landscapes. We have added a new supplementary figure (S13 Fig.) highlighting the probability density of the AF2 conformations projected onto the free energy landscapes, described in Results (p 9):

There is a remarkably good overlap between the AF-sampled ensemble of structures and the free-energy landscapes (Fig 5, S13 Fig).

We now also comment on the comparison between predicted state density and density of structures in the PDB; see response to 3.6 and Discussion (p 11) for details.

4.3. While I know this question might be out of scope for this study I would be curious to hear the authors comment/ add a paragraph in the discussion, if using the AF subsampled conformations as starting structures for MD simulations would be enough to sample and reconstruct the free energy surface obtained from aggregated 120 µs of sampling (in line with workflows shown in these following papers: https://pubs.acs.org/doi/10.1021/acs.jctc.2c01189, https://www.sciencedirect.com/science/article/abs/pii/S0969212624003708).

Response 4.3: We agree that it is intriguing to know whether AF-sampling can be combined with MD to reconstruct free energy landscapes, and now comment on this point in the Discussion; see response to 1.5 and Results (p 12) for details.

Once again, thank you for the many valuable comments and suggestions.

Sincerely,

Erik Lindahl & co-authors
