# Supplementary material for: Resolving the conformational ensemble of a membrane protein by integrating small-angle scattering with AlphaFold
Source: PLoS Comput Biol. 2025 Jun 27;21(6):e1013187. doi: 10.1371/journal.pcbi.1013187 (PMC12251176; doi:10.1371/journal.pcbi.1013187)
Supplement: Attachment — Submitted filename: lidbrink_response_signed.pdf [file pcbi.1013187.s019.pdf]

Stockholms universitet

Erik Lindahl
Professor of Biophysics

Stockholm May 9, 2025

Response to reviewer comments

Dear Dr. Slusky,

Thank you for the thoughtful consideration of our manuscript (PCOMPBIOL-D-24-01584). As demonstrated in the attached revision and outlined below, we believe we have responded to all remaining concerns of reviewers 1, 3, and 4, including edits to the Results, Discussion, and supplementary figure S6. In this response, reviewer comments are gray (renumbered for clarity), and quotes from the revised manuscript are written in red.

Sincerely,

Erik Lindahl and co-authors

Reviewer #1: The authors have addressed my concerns well. Congratulations on the nice manuscript.

Thanks for the kind words.

Reviewer #3: The authors have made changes to their manuscript in response to the reviewers comments and included ensemble, residuals, and real space comparisons, as requested. The current figures better visualize computational and experimental. On looking at the experimental vs prediction figures, gains made are minimal. Based on crystallographic RMSD, the authors have accurately predicted protein structures that are already deposited in the PDB. Based on SANS, the agreement experimental and models are open to interpretation and could be argued. The overall agreement of the curves to the models are good – as one would expect when the PDB includes the open and close structures, but the question is about differentiating two states. The authors write "The approach described above [comparison to SANS] enables accurate prediction of discrete conformation of GLIC." where discrete conformations are open and close states based on SANS data. Interpreting accuracy as matching the experimental data, the authors have not shown this clearly. Being able to accurately match predictions with solution data is an important problem, and the discussion section addresses this issue. However, the method here appears to be the first step and not the final one. Details are shown below.

3.1. The accuracy of predicted models to experimental data is not clear.
a. Figure 3 shows visible bias in the residuals. The similarity of 3A/B red and blue curves suggest that whatever is wrong is wrong in both sets of models.

R3.1.a: Thanks, good & thoughtful feedback. We do indeed see this as a first step to be further improved. The residuals indeed display visible bias, and we have modified the Results to explicitly comment on the similarity of the bias between the models (p. 7):

"To investigate the discrepancies in the fits from the AF-sampled conformations, we calculated the corresponding residuals, which exhibited a clear q-dependent bias that was similar for both models (Fig 3A, B)."

b. Figure 3C shows the difference between open and close for experimental and open and close for predicted, as requested by one of the reviewers. It is not clear to me the information value of this plot. As an analogy, 5000-4999=2-1.That doesn't mean 5000 and 2 are similar to each other.

Institutionen för biokemi och biofysik

Stockholms Universitet
Science for Life Laboratory
Box 1031
SE-171 21 Solna, Sweden

Besöksadress:
Science for Life Laboratory
Tomtebodavägen 23A, Solna

Tel: +46-734618050
Mobil: +46-734618050
E-post: erik.lindahl@dbb.su.se

Stockholms universitet

Erik Lindahl
Professor of Biophysics

Stockholm May 9, 2025

R3.1.b: This is a good comment. As many other SANS profiles these normalized, and since the low-q region primarily describes overall shape and the high-q region noise/bond vibration, SANS analysis is commonly focused on shifts in the mid-q region, in particular when we are interested in relative changes between states. We have modified the Results to clarify the purpose of the difference analysis (p. 6):

"Given that we were mainly interested in the relative changes between the two states, rather than absolute properties of either state, we focused our analysis on characterizing whether the difference between the predicted states could account for the observed difference between experimental conditions (Fig 3C). This allows characterization of potentially relevant substructure transitions, even in the context of possible systematic differences in computed vs. experimental models."

c. Figure 3D shows the chi square fit trying different ensembles, which is the closest I could find to a comparison between experimental and predicted. However, following a single metric that has bias for lower q does not justify the statement for "accurate prediction".

R3.1.c: The reviewer rightly points out imperfections in fits to our SANS data; although we acknowledge this in the Discussion (p. 11-12), we regret if the previous wording in Results seemingly suggested qualities of the models that we had not justified sufficiently. Instead, we now explicitly highlight the specific qualities we do have proper justification for, i.e. reproducibility and agreement with functional data. Results (p. 8):

"The approach described above enables reproducible predictions of discrete conformations of GLIC without using existing structural data. These predictions were consistent with functional data, with a closed state primarily corresponding to resting conditions and an open state associated with activating conditions."

d. Based on Fig. 3B, the major difference between open and close experimental is in the mid q range between 0.1 and 0,2, and this is where the emphasis should be placed.

R3.1.d: Strongly agree, and we appreciate the suggestion to emphasize this more; this is indeed where we found the largest difference between the predicted models. We now comment on this in the Results (p. 7):

"Encouragingly, the difference between the explicit-solvent SANS-curves was the largest at the scattering vectors where the experimental SANS curves differed the most (Fig 3A, B)."

e. Supplemental Fig 10 provides the real space calculation of the experimental and predicted. The experimental differences between open and close are subtle. By eye, the difference between the best predicted and either experimental curve is greater than the difference between experimental curves. The curves are difficult to distinguish, so if one peak is narrower than the other, it is difficult to judge.

R3.1.e: Agree - real-space curves are highly similar. The challenge is simply that the vast majority of pairwise distances in a protein do not change in a conformational transition, so the absolute values are dominated by other properties. Still, we believe it is valuable to show that the distribution shifts appear to be reproduced correctly, even if we were unable to find a representation that could isolate the differences more clearly. We have modified the Results to comment that the differences indeed are subtle (p. 7):

"Both the predicted models appeared to be modestly contracted relative to experimental SANS profiles, although the differences are subtle (S10 Fig)."

Stockholms universitet

Erik Lindahl
Professor of Biophysics

Stockholm May 9, 2025

f. It is difficult to conclude, based on the SANS analysis, that the authors were able to accurately predict discrete conformations. SANS could serve as a validation for the GLIC model, but not with regard to the open and close states. The authors may want to consider that this discrepancy between predicted and experimental may in part be due to the large pore even in the closed state and possible PEPSI-SAXS error in the the hydration layer prediction within that pore The authors may consider gromacs, which is reported to better handle hydration.

R3.1.f: Please see response 3.1.c for the statement on accuracy. As part of the work, we indeed found that explicit solvent methods (built on GROMACS) worked better to predict the SANS curves, and we regret if this was not clear. We now emphasize this in the Results, and comment in the Discussion

Results (p. 6):

“To more accurately capture subtle variations in the SANS curves, the explicit-solvent-based method WAXSiS implemented in GROMACS-SWAXS [35,36] was used to calculate SANS curves of the selected conformations (see Methods).“

Discussion (p. 11):

“The subtle gating transitions and a low open probability of GLIC only resulted in minor differences in the experimental SANS curves [27], but we could still robustly estimate the population shift between the experimental conditions that largely agreed with expectations (Fig 3). Given the subtlety of the differences, the usage of explicit-solvent SANS forward models may have been particularly important, especially since the vestibule is largely hydrated in both the closed and open states.”

3.2. For the crystallographic comparison, it is also difficult to see clearly. The overlays act like clouds in Fig 4 A, B, and E, and whether those clouds show differences between open and close is up for interpretation. The models have different pore sizes but does that reflect the pore size of the crystal is not obvious. The RMSD S11 table is the closest, but it is a singular value for an atomic structure. The most important aspect of the open and close is the pore size. It would be good to include a plot of the HOLE pore radius as a function of height (as viewed by A and B) for best predictions and crystal.

R3.2: We have now modified supplementary figure S6 and report pore profiles of predictions and crystal structures, and comment in the Results. To facilitate further analysis, all predicted atomic coordinates are also available on Zenodo.

Results (p. 8):

To examine whether the predictions reasonably represent the open and closed states, models were compared to crystal structures reported to represent closed (PDB ID 4NPQ) and open (PDB ID 4HFI) states of GLIC (Fig 4A, B, S6 Fig) [39]. The predicted conformations were consistent with corresponding crystal structures (with Cα RMSD values of 1.59 Å and 2.09 Å for the open and closed states respectively when aligning on the Cα-atoms), especially in the transmembrane domain (TMD) (with Cα RMSD values of 0.68 Å and 0.78 Å respectively). The pore radius profiles are highly similar for the closed states and they exhibit qualitatively similar expansions in the upper region (2.4 Å for predictions vs. 3.6 Å for crystal structures (S6 Fig).

S6 Fig. Pore profiles of predicted conformations and crystal structures of open (4HFI) and closed (4NPQ) states. Grey arrows indicate the maximal pore-radius expansions between the predicted conformations (solid) and the crystal structures (dashed). The pore profiles were calculated using CHAP [32].

3.3. With regard to potential influence from the PDB, the authors state that the prevalence of open

Stockholms universitet

Erik Lindahl
Professor of Biophysics

states in the PDB vs closed states in the AF prediction shows that AF is not biased. This reasoning doesn't make sense to me, 1) if AF is trained on the crystallographic database as a whole, which represents primarily lower energy states. The occurrence of closed states in the activated conditions, as determined in this paper, suggests that the closed state is the lower energy state. 2) How do you know that one example is sufficient for it to remember the relative relationship of residues in the closed state. To address this issue, the authors should consider adding in something like "we cannot exclude the possibility that AF is influenced by deposited PDBs of open and closed GLIC proteins"

R3.3: Agree; we cannot rule out that already deposited PDBs of GLIC may have influenced the AlphaFold predictions, and have modified the Discussion (p. 11) accordingly:

In the case of GLIC, it was possible to sample both open- and closed-like states. We cannot exclude the possibility that AF is influenced by deposited PDBs of open and closed GLIC proteins. Interestingly, however, AlphaFold sampled mostly closed-like conformations (Fig 4, S13 Fig), even though a substantial majority of GLIC structures in the PDB are open [41].

Reviewer #4: The authors have addressed all my comments. I recommend for publication!

Appreciated; thanks!
