# Supplementary material for: Resolving the conformational ensemble of a membrane protein by integrating small-angle scattering with AlphaFold
Source: PLoS Comput Biol. 2025 Jun 27;21(6):e1013187. doi: 10.1371/journal.pcbi.1013187 (PMC12251176; doi:10.1371/journal.pcbi.1013187)
Supplement: Attachment — Submitted filename: 241118reviewsaxsmembraneAF2.docx [file pcbi.1013187.s017.docx]

This paper, by Lidbrink and colleagues, tackles the complex problem of predicting distinct conformations of membrane proteins involved in signaling. It is an important problem whose solution would benefit multiple areas of health research. The authors innovatively combine a Jens Meilleur program to generate a diverse conformational set with their previously published experimental SANS data. They show that use of PCA analysis, they can generate models consistent with crystal structures of open and closed states. There is concern for potential “cheating” by AF3 with already deposited PDBs. The larger concern is that they state that their models are consistent with resting and activated states measured by SANS, which is not readily apparent in their figure. See discussion below.

1. I understand the part of using MSA sampling to generate different ensembles. On page 4 line 76, authors determined to remove conformations with a plDDT lower than 75. Are there any specific reasons for the cutoff? Will this cutoff remove flexible conformations from the prediction?
2. The authors state that their generated state assignments are consistent with the respective excited or resting state experimental data. From first glance at Figs. 3A and 3B, the models are consistent with the overall features of the experimental data. Yet, the goal of this work is to generate atomic predictions for individual states. Given that rigorous criteria, it is difficult to judge that the open or closed models agreed better to the experimental data than the other. Their residual shows a clear, q-dependent bias, indicating a significant difference between experimental and the atomic models. In this case, use of chi square could hide discrepancies. Could the authors explain based on the residuals how they reached their conclusion?
3. Comparing experimental with models in both reciprocal and real space plots can give distinct information and provide more intuitive insight into the difference.
4. For the experimental data, 1 or 2 sentences on how the data was collected would be relevant for this paper for assessing the efficacy of the methods. For example, was any size exclusion performed. How long was the data collection? What temperature? Concentration of ligand.
5. In Fig.3C, the authors try to use a linear combination of closed and open states to evaluate the prediction accuracy. Since a conformational ensemble usually does not consist of only two distinct conformations, is there a better way to evaluate the prediction result instead of simply using a linear combination of two PDBs? The authors did pathway analysis – could models along the pathway be used?
6. Additionally, it is the ensemble of closed and open structures that should be shown in comparison to the experimental data for Fig 3A.
7. The authors discussed the comparison between PDBs and predictions In the section starting from page 7 line 167. Clearly, there are some differences including the distribution of closed/open states and Fig.4 F. I suggest the author to further explain those differences. What does it mean that the proteins are primarily in the closed state?
8. The application of this method is on proteins with unknown structure. How would AF be influenced by structures already in the PDB, even if use of template is blocked? Has this been established for this work?
9. Do the models have proper geometry, based on Jane Richardson’s program Molprobity? If they do not, this can impact comparison to experimental data.
